# Polydopamine-Modified Metal–Organic Frameworks, NH_2_-Fe-MIL-101, as pH-Sensitive Nanocarriers for Controlled Pesticide Release

**DOI:** 10.3390/nano10102000

**Published:** 2020-10-10

**Authors:** Yongpan Shan, Chunli Xu, Hongjun Zhang, Huiping Chen, Muhammad Bilal, Shujun Niu, Lidong Cao, Qiliang Huang

**Affiliations:** 1 Institute of Plant Protection, Chinese Academy of Agricultural Sciences, No. 2 Yuanmingyuan West Road, Haidian District, Beijing 100193, China; shanyongpan@yeah.net (Y.S.); springxcl2013@126.com (C.X.); hpingchen@126.com (H.C.); mohammadentomologist@gmail.com (M.B.); 2 State Key Laboratory of Cotton Biology, Institute of Cotton Research, Chinese Academy of Agricultural Sciences, No. 38 Yellow River Avenue, Anyang 455000, China; 3 Institute for the Control of Agrochemicals, Ministry of Agriculture and Rural Affairs, Beijing 100125, China; hongjun-zh1975@163.com; 4 Institute of Plant Protection, Gansu Academy of Agricultural Sciences, No. 1 Nongkeyuan New Village, An’ning District, Lanzhou 730070, China; niu.shujun@163.com

**Keywords:** metal–organic framework, polydopamine, pesticide, pH-sensitive release, fungicidal activity

## Abstract

Recently, metal–organic frameworks (MOFs) have become a dazzling star among porous materials used in many fields. Considering their intriguing features, MOFs have great prospects for application in the field of sustainable agriculture, especially as versatile pesticide-delivery vehicles. However, the study of MOF-based platforms for controlled pesticide release has just begun. Controlled pesticide release responsive to environmental stimuli is highly desirable for decreased agrochemical input, improved control efficacy and diminished adverse effects. In this work, simple, octahedral, iron-based MOFs (NH_2_-Fe-MIL-101) were synthesized through a microwave-assisted solvothermal method using Fe^3+^ as the node and 2-aminoterephthalic acid as the organic ligand. Diniconazole (Dini), as a model fungicide, was loaded into NH_2_-Fe-MIL-101 to afford Dini@NH_2_-Fe-MIL-101 with a satisfactory loading content of 28.1%. The subsequent polydopamine (PDA) modification could endow Dini with pH-sensitive release patterns. The release of Dini from PDA@Dini@NH_2_-Fe-MIL-101 was much faster in an acidic medium compared to that in neutral and basic media. Moreover, Dini@NH_2_-Fe-MIL-101 and PDA@Dini@NH_2_-Fe-MIL-101 displayed good bioactivities against the pathogenic fungus causing wheat head scab (*Fusarium graminearum*). This research sought to reveal the feasibility of versatile MOFs as a pesticide-delivery platform in sustainable crop protection.

## 1. Introduction

Metal–organic frameworks (MOFs) are crystalline porous materials consisting of organic ligands coordinated to metal centers, and are recent innovations in the field of material chemistry [1,2]. MOFs have become a dazzling star among porous materials by virtue of their superior properties and promising applications in gas storage and separation [3,4], energy conversion and storage [5,6], water harvesting and splitting [7,8], heterogeneous catalysis [9,10], chemical sensors [11,12], environmental remediation [13,14], cancer therapy, drug delivery [15,16], etc. Considering their excellent performance, MOFs have great potential for application in the field of sustainable agriculture, especially as versatile pesticide-delivery vehicles. However, the study of MOF-based platforms for controlled pesticide release has just started.

Controlled pesticide release responsive to external environmental stimuli is highly desirable for decreased agrochemical input, improved control efficacy, and diminished adverse effects [17,18,19]. A lot of inorganic and organic materials have been widely used to prepare controlled-release formulations (CRFs) of pesticides [20,21]. Nevertheless, CRFs of pesticides based on MOFs have only a few reports. Yaghi et al. reported the synthesis of two MOFs based on Ca^2+^ ions and nontoxic, naturally occurring lactate and acetate linkers. These porous degradable Ca^2+^ MOFs can encapsulate an agricultural fumigant, *cis*-1,3-dichloropropene, exhibiting a release rate that is 100 times lower than that of liquid *cis*-1,3-dichloropropene [22]. Ethylene, a basic gaseous chemical, is an important exogenous plant hormone that regulates fruit ripening and senescence [23]. Guan et al. developed a novel matrix consisting of an aluminum-based MOF core and alginate-based shell, which was used for ethylene storage and controlled release using sodium citrate solution as a promoter [24]. Tang et al. reported the preparation of porous porphyrinic MOFs constructed from meso-tetra(4-carboxyphenyl)porphine as an organic linker and metal ion clusters, Zr (IV), as nodes, which were applied to load and control the release of the fungicide tebuconazole [25]. 

Among the plethora of MOF materials, iron-containing MOFs (Fe-MOFs) have great potential in practical applications due to their many favorable properties, such as biocompatibility, tunable pore size and surface area, diverse structure type, intriguing functionality, and preferable stability [26,27]. Moreover, iron is an indispensable micronutrient for crop growth and development, owing to its important role in chlorophyll synthesis in plants [28]. Thus, taking into account their multiple functions and atom economy, Fe-MOFs have considerable prospects for use as pesticide-delivery vehicles in sustainable plant protection. Recently, we prepared a simple octahedral Fe-MOF (Fe-MIL-100) constructed from trimers of iron linked by 1, 3, 5-benzenetricarboxylate. Azoxystrobin, as a model pesticide, was encapsulated into Fe-MIL-100 with a loading content of 16.24% [29]. Azoxystrobin-loaded Fe-MIL-100 exhibits good fungicidal activities against two pathogenic fungi causing wheat head scab and tomato late blight. However, the extent of the responsiveness to pH of the release is not satisfactory [29]. On the other hand, large initial burst releases at pH 7.2 and 8.5 were observed, which prevents an on-demand controlled and sustained release. To overcome this limitation, the surface modification of Fe-MOFs with diverse gatekeepers can provide solutions for controlled pesticide release in response to internal or external experimental stimuli.

Since the first report in 2007, as a mussel adhesive protein-inspired material, polydopamine (PDA) has emerged as one of the most powerful approaches to functionalizing virtually all material surfaces under mild conditions [30]. As the first single-step and material-independent surface chemistry process, PDA coating endows versatile materials with many intriguing features, especially a strong adhesive property [31,32]. PDA-modified MOFs have been reported and applied in biosensors [33], the removal of environmental pollutants [34,35,36], CO_2_ capture [37], and cancer therapy [38,39,40]. Considering the sensitivity of PDA to experimental stimuli [41,42,43], PDA-modified Fe-MOFs have great potential in delivering pesticides to address the limitations mentioned above.

As a systemic triazole fungicide, diniconazole (Dini) is widely used for the control of various fungi, particularly basidiomycetes and ascomycetes [44]. Diniconazole was thus chosen as a model pesticide to test the pH-responsive controlled release performance of PDA-modified Fe-MOFs. In the present study, amine-functionalized Fe-MOFs (NH_2_-Fe-MIL-101, MIL = Materials of Institut Lavoisier), prepared from 2-aminoterephthalic acid as an organic linker and FeCl_3_ as a metal source [45], were used as Dini carriers. The subsequent PDA modification endowed encapsulated Dini with a pH-responsive release profile and good fungicidal bioactivity against wheat head scab (*Fusarium graminearum*). The application scenario for the fungicide is complex, with varying pH values. The pH-responsive release of an active ingredient can potentially improve the utilization efficiency for a pesticide. This research sought to provide a novel method for the potential application of MOFs in the smart delivery of pesticides.

## 2. Materials and Methods

### 2.1. Materials

Iron (III) chloride hexahydrate (FeCl_3_·6H_2_O, 97%), dopamine hydrochloride and Tris-HCl were purchased from J&K Scientific Ltd. (Beijing, China). 2-aminoterephthalic acid (H_2_ATA) was purchased from Aladdin Reagent Co., Ltd. (Shanghai, China). Diniconazole (98%) was purchased from Beijing Green Agricultural Science and Technology Group Co., Ltd. (Beijing, China). The Pesticide Bioassay Lab in the Institute of Plant Protection of the Chinese Academy of Agricultural Sciences generously provided the wheat head scab fungus (*F. graminearum*). All other chemicals were commercially available and used without additional purification.

### 2.2. Synthesis of the Nanoparticles

#### 2.2.1. Synthesis of NH_2_-Fe-MIL-101 Nanocrystals

The synthesis of NH_2_-Fe-MIL-101 nanocrystals used a microwave irradiation method according to the procedure reported previously with a little modification [45]. Briefly, approximately 760.5 mg of H_2_ATA (4.2 mmol) and 2268 mg of FeCl_3_·6H_2_O (8.4 mmol) were dissolved in 210 mL of deionized water in a 500 mL round-bottom flask. The mixture was thereafter transferred into a Teflon-lined stainless autoclave, sealed and placed in a microwave oven (XH-800G). The autoclave was heated at 100 °C for 4 h by microwave irradiation at 400 W. The obtained NH_2_-Fe-MIL-101 was recovered by centrifugation at 10,000 rpm for 10 min. To remove the free acid, the nanocrystals were washed with fresh ethanol 3 times and dried under vacuum at 80 °C for further characterization and analysis.

#### 2.2.2. Preparation of Dini@NH_2_-Fe-MIL-101 Nanocrystals

Diniconazole was loaded into NH_2_-Fe-MIL-101 nanocrystals by a physical adsorption method. Generally, about 30 mg of Dini and 30 mg of NH_2_-Fe-MIL-101 nanocrystals were weighed in a 10 mL plastic centrifuge tube, and then, 1 mL of dichloromethane was added. Subsequently, the suspension was sealed and stirred at room temperature for 6 h. Diniconazole-loaded NH_2_-Fe-MIL-101 (denoted as Dini@NH_2_-Fe-MIL-101) was collected by centrifugation (10,000 rpm, 10 min) and drying at 50 °C.

To determine the loading content and encapsulation efficiency of Dini, approximately 5 mg of the prepared Dini@NH_2_-Fe-MIL-101 nanocrystals were suspended in 50 mL of methanol and extracted by ultrasonication for 3 h. Then, the concentration of the supernatant was measured by high-performance liquid chromatography (HPLC, 1200-DAD (Diode Array Detector), Agilent, Santa Clara, CA, USA). The HPLC operating conditions were as follows: ZORBAX SB-C_18_ reversed-phase column (5 µm, 4.6 × 150 mm); column temperature, 25 °C; mobile phase (methanol/0.1% formic acid aqueous solution (V/V) = 80:20); flow rate, 1.0 mL/min; injection volume, 5 μL; and detection wavelength, 220 nm. The loading content and encapsulation efficiency were calculated by the formulas reported by us [29].

#### 2.2.3. Preparation of Dopamine-Coated Dini@NH_2_-Fe-MIL-101 Nanocrystals

Dopamine-coated Dini@NH_2_-Fe-MIL-101 nanocrystals were prepared according to a previous procedure with a little modification [46]. Briefly, 720 mg of the as-prepared Dini@NH_2_-Fe-MIL-101 and 720 mg of dopamine hydrochloride were dispersed in 360 mL of Tris buffer solution (10 mM, pH = 8.5). The mixture was stirred at room temperature for 24 h. Afterwards, the resulting solid was separated by centrifugation (10,000 rpm, 10 min), washed with water and dried in an oven at 50 °C for 6 h to obtain the dopamine-coated Dini@NH_2_-Fe-MIL-101 (denoted as PDA@Dini@NH_2_-Fe-MIL-101).

### 2.3. Sample Characterization

The surface morphology of as-prepared nanoparticles was observed by scanning electron microscopy (SEM, FEI Quanta Q400, Eindhoven, Netherlands, operated at 20 kV). Powder X-ray diffraction analysis (XRD) was performed on a Bruker D8 Advance X-ray diffractometer (Bruker, Karlsruhe, Germany) with Cu Kα radiation (λ = 0.15418 nm). Data were collected in 2 theta of 5–30° with a step size of 0.02° at a scanning rate of 0.1°/s.

The elemental compositions of the samples were analyzed by X-ray photoelectron spectroscopy (XPS, Kratos Ltd., Manchester, UK) on a photoelectron spectrometer (ESCALab 250Xi, Thermo Fisher Scientific, Waltham, MA, USA) with 150 W monochromatic Al Kα radiation (1486.6 eV, 500 μm spot size) as the excitation source. The binding energies were calibrated by the C1s peak of the surface carbon at 284.8 eV. Energy-dispersive X-ray spectroscopy (EDS) mapping was further used to confirm the elemental composition.

The nitrogen adsorption/desorption isotherms and pore structure of the samples were studied with a specific surface area and pore size analyzer (TriStarII 3020, Micromeritics Instruments Corp, Norcross, GA, USA) at 77 K. The samples were degassed at 10^−3^ Torr and 120 °C for 6 h. The chemical structures of the samples were studied with a Fourier transform infrared spectrophotometer (FT-IR, Nicolet 6700, Thermo Fisher Scientific, Waltham, MA, USA) with a potassium bromide pellet. Thermogravimetric analyses (TGAs) were carried out using a PerkinElmer Pyris Diamond (Woodland, CA, USA) from 30 to 550 °C at 10 °C/min under a N_2_ atmosphere.

### 2.4. In Vitro Release of Dini

The controlled characteristics of Dini release from Dini@NH_2_-Fe-MIL-101 and PDA@Dini@NH_2_-Fe-MIL-101 were studied by a dialysis method in release medium containing phosphate buffered saline (PBS), ethanol and Tween-80 emulsifier (70:29.5:0.5, v/v/v). The pH-responsive release characteristics were studied in the release medium with different pH values (3.1, 7.0 and 10.3). Approximately 20 mg of pesticide-loaded nanoparticles were weighed in a dialysis bag (MW: 8000–14,000) with 5 mL of release medium. Then, the sealed dialysis bag was immersed in plastic bottles containing 195 mL of release medium and placed on a shaker with a speed of 100 rpm at 25 °C. At designated time intervals, 0.8 mL of the mixture was taken out for HPLC analysis. All the treatment was repeated three times. The accumulative diniconazole released was calculated according to our previous report [29].

### 2.5. Bioactivity Studies

The bioactivities of Dini@NH_2_-Fe-MIL-101 and PDA@Dini@NH_2_-Fe-MIL-101 were studied by the mycelium growth rate method. In this work, the wheat head scab fungus (*F. graminearum*) was selected as the tested fungus. A mycelial disc with a diameter of 5 mm was inoculated on potato dextrose agar plates. Before inoculation, the sterile molten potato dextrose agar was treated with diniconazole technical concentrate (TC), Dini@NH_2_-Fe-MIL-101 or PDA@Dini@NH_2_-Fe-MIL-101 under two different active-ingredient concentrations of 1 and 5 mg/L. Meanwhile, the bioactivities of the blank carrier for NH_2_-Fe-MIL-101 and control check (CK) without any treatment were also analyzed. Each treatment was repeated five times. After 4 days of incubation at 25 °C, the colony diameter was measured by the cross method and the biological activity is expressed as the percentage of inhibition (%), which was calculated as equal to (colony diameter of control – colony diameter of treatment)/(colony diameter of control – diameter of mycelial discs) × 100.

### 2.6. Statistical Analysis

One-way analysis of variance (ANOVA) and Duncan’s multiple range tests were performed on the data using the SPSS 10.0 software (SPSS, Chicago, IL, USA). The confidence intervals used in this study were based on 95% (*p* < 0.05). All data are plotted as mean ± standard error.

## 3. Results and Discussion

### 3.1. Preparation and Characterization of Nanoparticles

Metal-organic frameworks (MOFs) are a class of crystalline micro-mesoporous hybrid materials. Recently, they have shown potential applications in pesticide-delivery systems due to their high specific surface area and uniform-but-tunable cavities. In the current study, we prepared amino-modified MOFs (NH_2_-Fe-MIL-101) through a rapid microwave-assisted solvothermal synthesis method according to the procedure reported by Horcajada et al. [45]. In fact, most of the references reported the preparation of this MOF by the solvothermal method using conventional heating and dimethyl formamide (DMF) as a solvent, which is adapted from the procedure reported by Bauer et al. [47]. However, when microwave irradiation was used, water instead of DMF was used as the solvent, which is environmentally friendly. Moreover, the reaction time was remarkably shortened from 24 h to 4 h. Thus, a microwave-assisted solvothermal method was adopted to prepare NH_2_-Fe-MIL-101, which was used as a carrier for loading the pesticide diniconazole to afford Dini@NH_2_-Fe-MIL-101. The PDA was thereafter coated on the surface of Dini@NH_2_-Fe-MIL-101 to obtain PDA@Dini@NH_2_-Fe-MIL-101.

**Figure 1:**
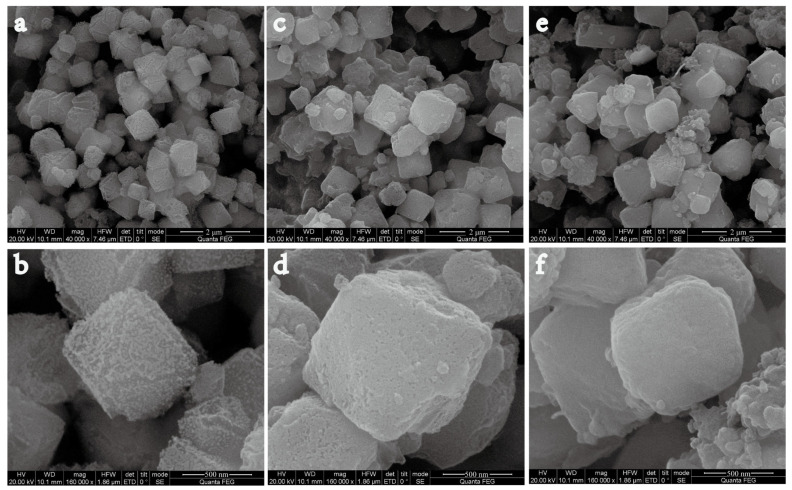
SEM images of the NH_2_-Fe-MIL-101 (**a**,**b**), Dini@NH_2_-Fe-MIL-101 (**c**,**d**) and PDA@Dini@NH_2_-Fe-MIL-101. Scale length: (**a**,**c**,**e**) 2 μm; (**b**,**d**,**f**) 500 nm.

**Figure 2:**
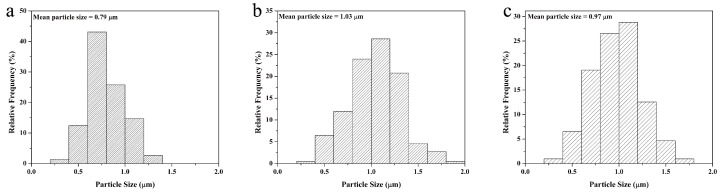
The distribution of particle sizes of NH_2_-Fe-MIL-101 (**a**), Dini@NH_2_-Fe-MIL-101 (**b**) and PDA@Dini@NH_2_-Fe-MIL-101 (**c**) based on 200 specimens.

The morphology of the as-prepared nanoparticles was observed using SEM. The SEM micrographs showed that NH_2_-Fe-MIL-101 (Figure 1a,b), Dini@NH_2_-Fe-MIL-101 (Figure 1c,d) and PDA@Dini@NH_2_-Fe-MIL-101 (Figure 1e,f) are all octahedrons with regular shapes and homogeneous particle sizes. The surfaces of Dini@NH_2_-Fe-MIL-101 became relatively smooth after loading Dini. After PDA coating, the surfaces of PDA@Dini@NH_2_-Fe-MIL-101 became smoother. The average diameters of the samples were determined by statistical analysis of the SEM images of 200 randomly selected particles. The average diameters of NH_2_-Fe-MIL-101, Dini@NH_2_-Fe-MIL-101 and PDA@Dini@NH_2_-Fe-MIL-101 are 0.79, 1.03 and 0.97 μm, respectively (Figure 2). The average diameters increased slightly after Dini loading and PDA coating.

**Figure 3:**
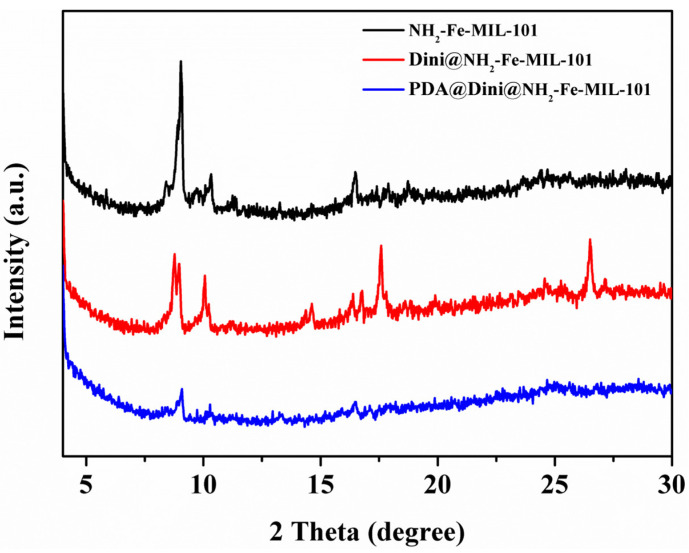
XRD pattern of NH_2_-Fe-MIL-101.

The crystallographic structures of the samples were determined by powder XRD. The XRD pattern is crucial for confirming the formation of NH_2_-Fe-MIL-101. The exact structure of the MOF is quite important in view of the polymorphism effect, and the properties of polymorphs depend on the synthetic conditions, even if the same components are used. Recently, Dong et al. found that the conventional solvothermal and microwave-assisted methods have obvious influences on the XRD patterns of NH_2_-Fe-MIL-101, and fewer peaks were observed when microwave irradiation was used [48]. The XRD patterns of NH_2_-Fe-MIL-101, Dini@NH_2_-Fe-MIL-101 and PDA@Dini@NH_2_-Fe-MIL-101 are presented in Figure 3. In the present study, the NH_2_-Fe-MIL-101 was prepared using microwave irradiation. The instantaneous microwave energy causes a reduction in crystallinity [48]. As a result, NH_2_-Fe-MIL-101 shows weaker and fewer diffraction peaks that are consistent with those prepared with a similar method [48]. However, when the conventional solvothermal method was used, more and more-intense diffraction peaks were observed [49,50]. After the loading of Dini, new peaks were found, which were possibly attributable to a Dini salt or some contamination that is washed away after the PDA coating. Successful PDA coating further led to a loss of crystallinity.

**Figure 4:**
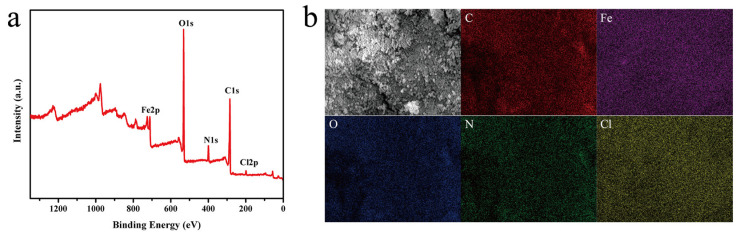
XPS spectra of NH_2_-Fe-MIL-101 (**a**), SEM images of NH_2_-Fe-MIL-101 and the corresponding EDS-elemental mapping of C, Fe, O, N and Cl (**b**).

X-ray photoelectron spectroscopy (XPS) can provide accurate information about the elements on the surface of the sample. The XPS spectra of NH_2_-Fe-MIL-101 are presented in Figure 4a. The binding energies were at approximately 284.8, 711.9, 531.8, 399.5 and 198.5 eV and belonged to C1s, Fe2p, O1s, N1s and Cl2p, respectively. The EDS mapping spectrum of NH_2_-Fe-MIL-101 also clearly reveals the presence of carbon, iron, oxygen, nitrogen and chlorine elements (Figure 4b).

**Figure 5:**
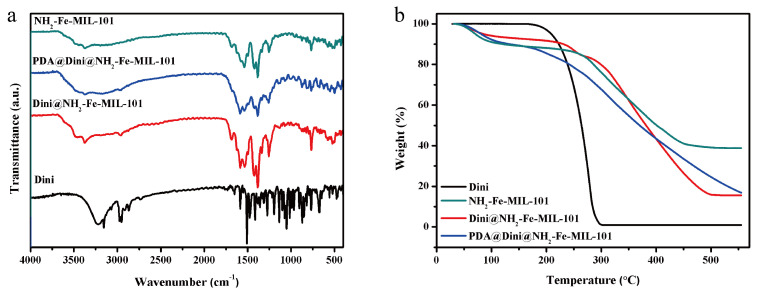
FTIR spectra (**a**) and TGA (**b**) of Dini, NH_2_-Fe-MIL-101, Dini@NH_2_-Fe-MIL-101 and PDA@Dini@NH_2_-Fe-MIL-101.

The FTIR spectra of the products are shown in Figure 5a. The characteristic absorption peak at 3368 cm^−1^ is attributed to the asymmetrical and symmetrical stretching vibration of the amine groups in NH_2_-Fe-MIL-101. The peak at 1581 cm^−1^ is associated with the C=N bonding of NH_2_-Fe-MIL-101. Diniconazole exhibits characteristic absorption at 2955 cm^−1^ corresponding to the C–H stretching vibration, which can be observed in Dini@NH_2_-Fe-MIL-101 and PDA@Dini@NH_2_-Fe-MIL-101, indicating the successful loading of Dini into NH_2_-Fe-MIL-101.

TGA is frequently used to study the thermal stability and decomposition pattern of chemicals and materials. The curves for the Dini, NH_2_-Fe-MIL-101, Dini@NH_2_-Fe-MIL-101 and PDA@Dini@NH_2_-Fe-MIL-101 are depicted in Figure 5b. The material weight loss before 150 °C was probably due to the volatilization of vapor in the sample. The occurrence of a large weight loss between 150 and 550 °C might be due to the decomposition of the NH_2_-Fe-MIL-101 structure. More weight was lost by Dini@NH_2_-Fe-MIL-101 than NH_2_-Fe-MIL-101, indicating the successful loading of Dini. The total weight losses of NH_2_-Fe-MIL-101 and Dini@NH_2_-Fe-MIL-101 in the range 150–550 °C were approximately 50.1% and 76.6%, respectively. The difference in weight loss of 26.6% should be the loading content of Dini in NH_2_-Fe-MIL-101. The loading content was also measured by an HPLC method, and the result was determined to be 28.1%, which was largely consistent with TGA result.

**Figure 6:**
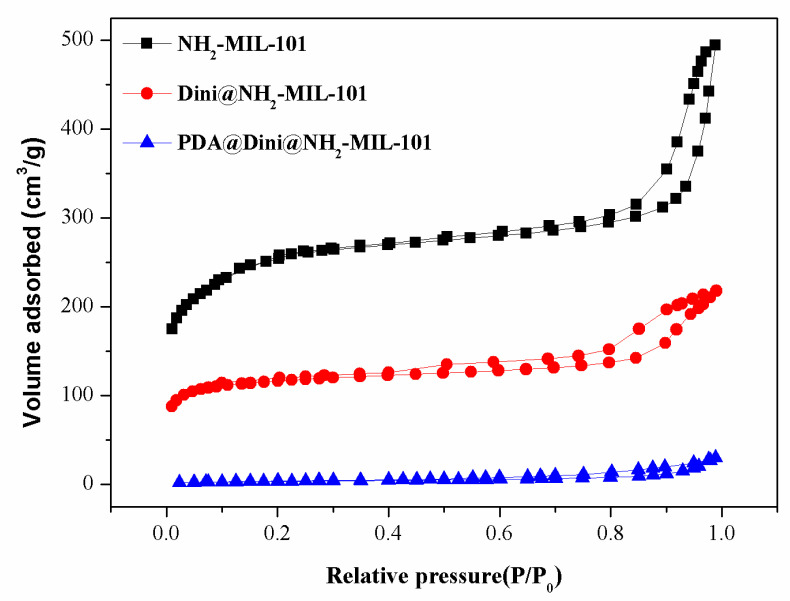
Nitrogen adsorption–desorption isotherms of NH_2_-Fe-MIL-101, Dini@NH_2_-Fe-MIL-101 and PDA@Dini@NH_2_-Fe-MIL-101.

The porous structures of the as-prepared MOFs were examined according to the Brunauer–Emmett–Teller (BET) specific surface area and Barrett–Joyner–Halenda (BJH) pore size and volume analyses. Figure 6 shows the N_2_ adsorption–desorption isotherms of NH_2_-Fe-MIL-101, Dini@NH_2_-Fe-MIL-101 and PDA@Dini@NH_2_-Fe-MIL-101. Table 1 summarizes the values of the BET specific surface area (S_BET_), the total pore volume (V_t_) and the BJH pore diameter (D_BJH_) of the samples. After the loading of Dini, the pores of NH_2_-Fe-MIL-101 were filled, and the S_BET_ and V_t_ were reduced from 953.9 to 449.8 m^2^/g and from 0.77 to 0.34 cm^3^/g, respectively, suggesting that most of the micropores were occupied by the Dini molecules. The pores of NH_2_-Fe-MIL-101 were further filled after PDA coating, and the S_BET_ and V_t_ were reduced from 449.8 to 13.0 m^2^/g and from 0.34 to 0.05 cm^3^/g, respectively, suggesting that PDA was successfully coated on the surface of Dini@NH_2_-Fe-MIL-101.

### 3.2. Loading of Dini into NH_2_-Fe-MIL-101 Nanoparticles

The loading content (LC) and encapsulation efficiency (EE) of Dini were optimized by adjusting the solvent and pesticide–carrier mass ratio; the results under various conditions are summarized in Table 2 for the loading test. Under the pesticide–carrier mass ratio of 1:1, when different solvents including acetone, methanol and dichloromethane were used, the LC was 24.9%, 24.3% and 28.1%, respectively. As the solvent would affect the LC, dichloromethane was selected as the solvent to load Dini for further optimization. As expected, the LC increased with an increasing pesticide–carrier ratio, possibly because of the higher Dini concentration, which promotes the carrier’s adsorption of pesticide molecules. However, the EE gradually decreased. Considering the LC and EE together, the large-scale preparation of Dini@NH_2_-Fe-MIL-101 samples was performed with a pesticide–carrier ratio of 1:1 for sample characterization, PDA modification, controlled release and bioactivity assays. After PDA coating, the LC was determined to be 14.7% due to the introduction of PDA.

### 3.3. pH-Sensitive Release

**Figure 7:**
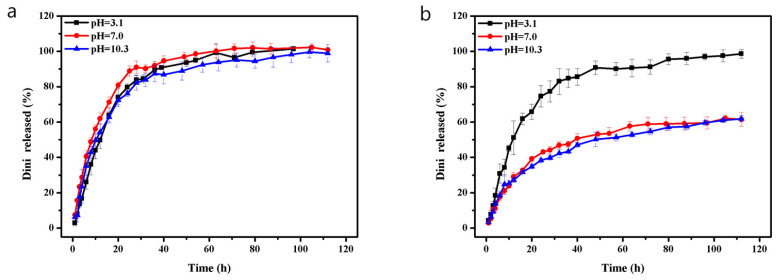
The curves of Dini release from Dini@NH_2_-Fe-MIL-101 (**a**) and PDA@Dini@NH_2_-Fe-MIL-101 (**b**) at different pH values of 3.1, 7.0 and 10.3. Error bars correspond to standard errors of triplicate measurements.

Polydopamine microcapsules and PDA-modified materials have been reported for controlled pesticide release because of the pH-responsive characteristic of PDA [30,51,52]. The profiles of Dini release from Dini@NH_2_-Fe-MIL-101 and PDA@Dini@NH_2_-Fe-MIL-101 are shown in Figure 7. In this study, a mixture solution, at three different pH values, of PBS, ethanol and Tween-80 emulsifier (70:29.5:0.5, v/v/v) was adopted as the release medium. The release rate of Dini@NH_2_-Fe-MIL-101 was faster than that of PDA@Dini@NH_2_-Fe-MIL-101 at three different pH values. The amount of Dini released from Dini@NH_2_-Fe-MIL-101 showed no obvious difference with different pH values (Figure 7a). However, the release of Dini from PDA@Dini@NH_2_-Fe-MIL-101 was pH-sensitive (Figure 7b). At pH 3.1, the accumulative release reached nearly 97% after 100 h, whereas at pH 7.0 and 10.3, the corresponding release values reached only 61%.

The self-polymerization of dopamine on the surface of NH_2_-Fe-MIL-101 occurs in neutral and basic media to form an adherent polymer coating [30]. The PDA coating could block the pores and confine Dini molecules inside the pores of NH_2_-Fe-MIL-101 in neutral and basic conditions, which is definitely beneficial for avoiding initial burst release [41]. In acidic media, however, the PDA coating might be partially peeled off from the surface of NH_2_-Fe-MIL-101, resulting in a faster release compared to that in neutral or basic media.

### 3.4. Bioassay of Dini-Loaded Nanoparticles

**Figure 8:**
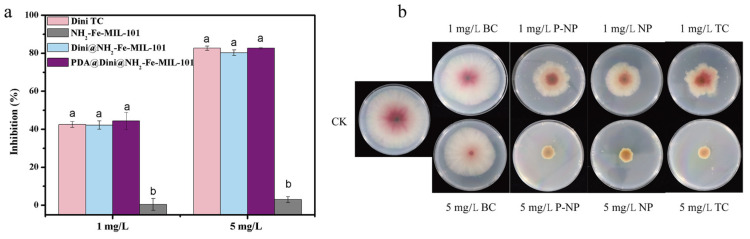
The histograms (**a**) and colony images (**b**) of the fungicidal activity of Dini technical concentrate (TC), Dini@NH_2_-Fe-MIL-101 (NP), PDA@Dini@NH_2_-Fe-MIL-101 (P-NP) and blank carrier of NH_2_-Fe-MIL-101 (BC) against the wheat head scab fungus (*F. graminearum*) at 4 days. Error bars correspond to standard errors of triplicate measurements. Bars marked with different letters are statistically different at *p* ≤ 0.05 as determined by Duncan’s multiple range tests.

The fungicidal activities of Dini@NH_2_-Fe-MIL-101 and PDA@Dini@NH_2_-Fe-MIL-101 were determined by the mycelium growth rate method. The control efficiencies for wheat head scab (*F. graminearum*) at two different concentrations, 1 and 5 mg/L, are presented in Figure 8a, and the images of colonies are shown in Figure 8b. The bioactivities of the blank carriers of NH_2_-Fe-MIL-101 and Dini technical concentrate (TC) were also tested as controls. After 4 days of incubation at 25 °C, the inhibition by Dini TC at concentrations of 1 and 5 mg/L was found to be 43% and 83%, respectively. The corresponding inhibition by Dini@NH_2_-Fe-MIL-101 and PDA@Dini@NH_2_-Fe-MIL-101 was (42% and 80%) and (44% and 83%), respectively. The findings clearly indicate that Dini@NH_2_-Fe-MIL-101 and PDA@Dini@NH_2_-Fe-MIL-101 have fungicidal bioactivity against *F. graminearum* that is comparable to that of Dini TC.

In the present study, the testing of the fungicidal activity against *F. graminearum* by the plate method was mainly to demonstrate the effectiveness of the as-prepared nano-delivery system, which could not clearly explain the effect of different pHs on controlled release. However, the concept and developed method for controlled pesticide release will find wide application in agricultural practice. The optimal way is to perform the field trails under real application scenarios to demonstrate the relationship between bioactivity and controlled release; this will be the future direction of our research.

## 4. Conclusions

In this study, porous NH_2_-Fe-MIL-101 was synthesized through a rapid microwave-assisted solvothermal synthesis using Fe^3+^ as the node and H_2_ATA as the organic ligand. Diniconazole, as a model fungicide, was loaded into NH_2_-Fe-MIL-101 by a physical absorption method. Under the optimized conditions of a mass ratio of the pesticide to carrier of 1:1 and using dichloromethane as the solvent, the LC and EE were 28.06% and 40.75%, respectively. The subsequent PDA modification could endow PDA@Dini@NH_2_-Fe-MIL-101 with pH-sensitive release patterns. The release of Dini from PDA@Dini@NH_2_-Fe-MIL-101 was much faster in an acidic medium than that in neutral and basic media. Compared with Dini TC, Dini@NH_2_-Fe-MIL-101 and PDA@Dini@NH_2_-Fe-MIL-101 displayed comparable fungicidal bioactivity against the pathogenic fungus *F. graminearum*. This research revealed the feasibility of versatile Fe-MOFs as a pesticide-delivery platform in sustainable crop protection.

## Figures and Tables

**Table 1:** Mesoporous structure characterization of nanoparticles ^a^.

Sample	S_BET_ (m^2^/g)	V_t_ (cm^3^/g)	D_BJH_ (nm)
NH_2_-Fe-MIL-101	953.9	0.77	3.2
Dini@NH_2_-Fe-MIL-101	449.8	0.34	3.0
PDA@Dini@NH_2_-Fe-MIL-101	13.0	0.05	-

^a^ S_BET_, Brunauer–Emmett–Teller (BET) specific surface area; V_t_, total pore volume; D_BJH_, Barrett–Joyner–Halenda (BJH) pore diameter.

**Table 2:** Loading content (LC) and encapsulation efficiency (EE) of diniconazole in NH_2_-Fe-MIL-101 with different solvents and mass ratios of pesticide to carrier ^a.^

Entry	Solvent	Mass Ratio	LC (%)	EE (%)
1	acetone	1	24.9 ± 0.5	33.6 ± 2.1
2	methanol	1	24.3 ± 0.1	32.3 ± 1.1
3	dichloromethane	1	28.1 ± 0.1	40.8 ± 0.4
4	dichloromethane	0.5	23.3 ± 0.2	60.9 ± 1.7
5	dichloromethane	2	30.9 ± 0.1	23.1 ± 0.5
6	dichloromethane	3	35.8 ± 0.2	19.5 ± 0.2
7	dichloromethane	4	43.0 ± 0.2	19.3 ± 0.3

^a^ Values are mean ± SD of three replicates.
